# The prognostic implications of podoplanin in cancer‐associated fibroblasts and PD‐L1 expression in high‐grade neuroendocrine carcinoma of the lung

**DOI:** 10.1111/1759-7714.15477

**Published:** 2024-11-02

**Authors:** Tatsuya Miyamoto, Tomohiro Haruki, Karen Makishima, Shinji Matsui, Yuki Oshima, Yoshihisa Umekita, Hiroshige Nakamura

**Affiliations:** ^1^ Department of Surgery, Division of General Thoracic Surgery, Faculty of Medicine Tottori University Tottori Japan; ^2^ Department of Pathology, Division of Pathology, Faculty of Medicine Tottori University Tottori Japan

**Keywords:** cancer‐associated fibroblast, podoplanin, programmed death‐ligand 1

## Abstract

**Objectives:**

Podoplanin (PDPN) expression in cancer‐associated fibroblasts (CAFs) (CAF‐PDPN) is considered a poor prognostic factor in nonsmall cell lung cancer, but little is known about its clinical significance in high‐grade neuroendocrine carcinoma of the lung (HGNEC). This study examines the association between CAF‐PDPN and stromal programmed death‐ligand 1 (PD‐L1) expression and the prognostic implications of CAF‐PDPN and PD‐L1 expression status in surgically resected HGNEC patients.

**Methods:**

Immunohistochemical analyses were performed on 121 resected HGNEC specimens using antibodies against PDPN and PD‐L1. Correlations between CAF‐PDPN, stromal PD‐L1 expression, and clinicopathologic features and their implications for survival were analyzed statistically.

**Results:**

There were substantially more large‐cell neuroendocrine carcinomas in the stromal PD‐L1‐positive group and more vascular invasion in the tumoral PD‐L1‐positive group. PDPN expression in CAF was moderately correlated with stromal PD‐L1 expression (*ρ* = 0.567, *p* < 0.001). In a survival analysis combining CAF‐PDPN and stromal PD‐L1 status, the 5‐year RFS rates for Group A: CAF‐PDPN (+)/stromal PD‐L1 (+), Group B: CAF‐PDPN (+)/stromal PD‐L1 (−), Group C: CAF‐PDPN (−)/stromal PD‐L1 (+), and Group D: CAF‐PDPN (−)/stromal PD‐L1 (−) were 62.0%, 46.8%, 17.5%, and 20.2%, respectively, with corresponding 5‐year OS rates of 76.6%, 69.2%, 27.0%, and 25.3%. The log‐rank test showed statistically significant differences among the groups in RFS (*p* < 0.001) and OS (*p* < 0.001).

**Conclusions:**

There is a correlation between CAF‐PDPN and tumoral/stromal PD‐L1 expression, and positive status for either CAF‐PDPN or stromal PD‐L1 expression could be an independent favorable prognostic factor in surgically resected HGNEC patients.

## Graphical abstract

We analyzed the correlation between CAF‐PDPN and stromal PD‐L1 expression and their implications for survival in patients with surgically resected high‐grade neuroendocrine carcinoma of the lung (HGNEC). There is a correlation between CAF‐PDPN and tumoral/stromal PD‐L1 expression, and positive status for either CAF‐PDPN or stromal PD‐L1 expression could be an independent favorable prognostic factor in surgically resected HGNEC patients.

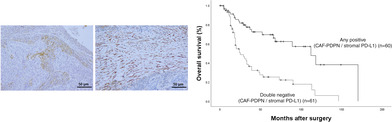

## INTRODUCTION

High‐grade neuroendocrine carcinoma (HGNEC) is composed of small‐cell lung cancer (SCLC) and large‐cell neuroendocrine carcinoma (LCNEC), which account for 15% and 2%–3% of all lung cancers, respectively.^1^, ^2^, ^3^ HGNEC may be curable in early stages, with surgical resection and adjuvant chemotherapy.^4^, ^5^ However, the majority of HGNECs are in an advanced stage at the time of diagnosis and have a very poor prognosis, with a 5‐year overall survival (OS) rate of <15% for SCLC and 15%–25% for LCNEC.^3^ In recent years, subtype classifications for SCLC and LCNEC have been identified. SCLC is classified into four subtypes based on ASCL1, NEUROD1, POU2F3, and YAP1 expression patterns.^6^ LCNEC is classified into two subtypes, SCLC‐like and nonsmall cell lung cancer (NSCLC)‐like subsets, based on genomic profiling using next‐generation sequencing.^7^ Although the molecular and pathological characteristics of HGNEC are gradually becoming more apparent, the prognosis has not yet improved compared with NSCLC.^8^ In recent years, triple therapy combining two conventional cytotoxic anticancer agents plus an immune checkpoint inhibitor (ICI) has been recognized as effective, and it has become the standard first‐line treatment for advanced SCLC.^9^ However, the effect of ICI on SCLC is limited, and it is presumed that the tumor microenvironment (TME) plays a major role. Therefore, elucidating the dynamics of cancer‐associated fibroblasts (CAFs) and immune cells, key cell types in the TME, will help develop new therapies and overcome ICI resistance. Kubouchi et al. and Yurugi et al. focused on podoplanin (PDPN), a CAF marker, and reported that overexpression of PDPN in CAF has a negative prognostic impact in NSCLC.^10^, ^11^ Although the dynamics of CAF overexpressing PDPN are becoming clear,^12^ there are few reports of the prognostic implications of CAF overexpressing PDPN in HGNEC.^13^ In this study, we performed immunohistochemical analysis of PDPN expression in CAF (CAF‐PDPN), programmed death‐ligand 1 (PD‐L1) expression in the tumor (tumoral PD‐L1), and PD‐L1 expression in the stroma (stromal PD‐L1). PD‐L1 is an effect predictor bio‐marker of ICI efficacy. We then evaluated the correlation and prognostic implications of these markers in surgically resected HGNEC patients.

## METHODS

### Patients and tumor specimens

**FIGURE 1:**
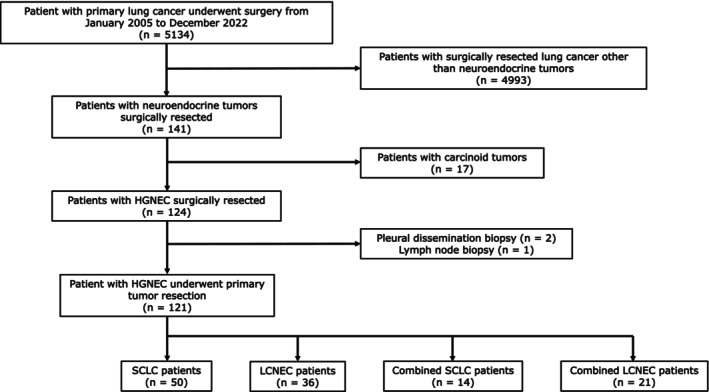
Flowchart of the selection of patients for inclusion in this study.

We collected 124 tissue samples of HGNEC surgically resected at Tottori University Hospital and four other affiliated hospitals (Tottori Prefectural Central Hospital, Tottori Prefectural Kousei Hospital, Yonago Medical Center, and Matsue Medical Center) between January 2005 and December 2022. Three patients were excluded because of pleural dissemination biopsy (*n* = 2) and lymph node biopsy (*n* = 1). Therefore, 121 cases with HGNEC were enrolled in our study. Figure 1 illustrates the study selection process. Pathological diagnosis was performed by pathologists in all cases according to the current World Health Organization classification criteria^14^ and the 8th edition of the TNM classification of lung cancer.^15^ LCNEC was defined as nonsmall cell carcinoma with neuroendocrine morphology (organoid nesting, palisading, rosettes, and trabeculae), high mitotic count (>10 mitoses/2 mm^2^), and positive immunohistochemical staining for one or more neuroendocrine markers (chromogranin A, synaptophysin and CD56).^14^ In this study, neuroendocrine markers were stained in 79 of 121 cases. In 7 of the 57 LCNEC and combined LCNEC, IHC was either not performed or unknown whether it was performed. Clinicopathological data for the patients were obtained from medical records. This study was approved by the ethics committee of the Faculty of Medicine, Tottori University, in May 2020 (20A002) and by the certified review board of each participating institution.

### Immunohistochemistry

Immunohistochemistry (IHC) staining was performed with a BenchMark GX (Ventana Medical Systems) or a Histostainer‐36A (Nichirei Biosciences). The primary antibodies used were anti‐PDPN (D2‐40/760‐4395, Roche Diagnostics) and anti‐PD‐L1 (E1L3N/13684, Cell Signaling Technology; 1:400). Formalin‐fixed and paraffin‐embedded specimens were cut into 4‐μm‐thick sections. PDPN IHC staining was performed with a BenchMark GX (Ventana Medical Systems). Sections were deparaffinized using EZ Prep (Ventana Medical Systems) and Cell Conditioning 1. Subsequently, sections were incubated with anti‐PDPN for 32 min. An OptiView DAB IHC Detection Kit (Ventana Medical Systems) was used to visualize the bound primary antibody. Finally, slides were counterstained with Hematoxylin II (Ventana Medical Systems) for 8 min and Bluing Reagent (Ventana Medical Systems) for 4 min. PD‐L1 IHC staining was performed with a Histostainer‐36A (Nichirei Biosciences). Sections were deparaffinized, hydrated, and incubated in a heat processor solution, pH 9 (Nichirei Biosciences), for 10 min at 100°C. Sections were blocked with 3% H_2_O_2_ (Nichirei Biosciences) for 5 min and incubated with anti‐PD‐L1 for 30 min. Subsequently, slides were incubated with a secondary antibody MAX‐PO (MULTI) (Nichirei Biosciences) for 30 min, visualized with DAB (Nichirei Biosciences), and counterstained with hematoxylin.

### Evaluation of immunohistochemical staining

We defined “cancer‐associated fibroblasts (CAFs)” as all fibroblasts present in the stroma within the tumor area, regardless of whether they were adjacent to cancer cells or not. CAF‐PDPN was defined as positive when the percentage of area of PDPN‐positive CAF in the tumor stromal area was ≥10%. Unlike our previous study,^10^, ^11^ the evaluation was based on area percentage rather than cell percentage. The population area was defined as the area of the tumor stroma, and the area was calculated visually. Lymphatic vessels were excluded from the PDPN‐positive area, but were included in the population area. For PD‐L1 staining, the tumor cells and tumor‐infiltrating immune cells in the stroma were evaluated. Tumoral PD‐L1 was defined as positive when ≥1% of the cells stained positive, and stromal PD‐L1 was defined as positive when ≥10% of the cells stained positive.^16^ All slides were evaluated independently by the author (T.M.) and the qualified pathologist (K.M.), who were blinded to the clinicopathological data. When different interpretations were obtained, slides were reviewed until a consensus was obtained.

### Statistical analysis

All statistical analyses were performed using SPSS version 29 (IBM SPSS Statistics; IBM Corporation). The correlation between CAF‐PDPN, tumoral PD‐L1, stromal PD‐L1 expressions, and clinicopathological factors was evaluated using the chi‐squared test. Spearman's rank correlation coefficient was used to assess correlations between CAF‐PDPN and tumoral PD‐L1 expression and between CAF‐PDPN and stromal PD‐L1 expression. We estimated the recurrence‐free survival (RFS) and overall survival (OS) for the survival analysis. RFS was defined as the period from the date of surgery to the date of recurrence or death from any cause. OS was defined as the period from the date of surgery to the date of death from any cause. Patients who were alive were censored at the time of their last follow‐up visit. Survival curves were computed using the Kaplan–Meier method, and differences in RFS and OS were analyzed using the log‐rank test. The Cox regression hazard model was used to evaluate the effect of various factors on RFS and OS to determine the independent prognostic value. All tests were two‐sided, and *p* < 0.05 was considered to be statistically significant in all tests.

## RESULTS

### Patient characteristics

The clinicopathological characteristics of the 121 cases with HGNEC are summarized in Table 1. The median age was 72 (range, 50–90) years; 109 (90.1%) patients were male, and 107 (88.4%) patients were heavy smokers (≥30 pack years). Surgical procedures included lobectomy (59.5%), segmentectomy (6.6%), wedge resection (28.9%), bilobectomy (4.1%), and pneumonectomy (0.8%). The pathological stage was Stage I in 75 (62.0%), Stage II in 23 (19.0%), Stage III in 18 (14.9%), and Stage IV in 5 (4.1%) cases. The histological diagnosis was SCLC in 50 (41.3%), LCNEC in 36 (29.7%), combined SCLC in 14 (11.6%), and combined LCNEC in 21 (17.4%) cases. Adjuvant chemotherapy was administered to 66 (54.5%) patients. Among these patients, 55 patients received cisplatin or carboplatin with etoposide. Patients in the stromal PD‐L1 expression positive group were classified with more LCNEC and fewer SCLC than those in the stromal PD‐L1 expression negative group (*p* = 0.022). Tumors from patients in the tumoral PD‐L1 expression positive group exhibited more vascular invasion than those in the tumoral PD‐L1 expression negative group (*p* = 0.041). There were no statistically significant differences between the groups for other clinicopathological factors.

**TABLE 1:** Clinicopathological characteristics of patients.

Characteristic		Tumoral PD‐L1 expression			Stromal PD‐L1 expression			CAF‐PDPN expression		
	Total (*n* = 121)	Positive (*n* = 20)	Negative (*n* = 101)	*p‐*value	Positive (*n* = 46)	Negative (*n* = 75)	*p‐*value	Positive (*n* = 50)	Negative (*n* = 71)	*p‐*value
Age (year, median, range)	72 (50–90)	69 (55–81)	72 (50–90)		71 (50–88)	73 (54–90)		72 (50–88)	72 (54–90)	
<70	46 (38.0)	10 (50.0)	36 (35.6)	0.227	19 (41.3)	27 (36.0)	0.560	18 (36.0)	28 (39.4)	0.701
≥70	75 (62.0)	10 (50.0)	65 (64.4)		27 (58.7)	48 (64.0)		32 (64.0)	43 (60.6)	
Gender										
Male	109 (90.1)	19 (95.0)	90 (89.1)	0.421	43 (93.4)	66 (88.0)	0.328	46 (92.0)	63 (88.7)	0.554
Female	12 (9.9)	1 (5.0)	11 (10.9)		3 (6.5)	9 (12.0)		4 (8.0)	8 (11.3)	
Smoking status										
<30 pack years	13 (10.7)	1 (5.0)	12 (11.9)	0.454	3 (6.5)	10 (13.3)	0.359	2 (4.0)	11 (15.5)	0.583
30–60 pack years	70 (57.9)	14 (70.0)	56 (55.4)		30 (65.2)	40 (53.3)		33 (66.0)	37 (52.1)	
>60 pack years	37 (30.6)	5 (25.0)	32 (31.7)		13 (28.3)	24 (26.7)		15 (30.0)	22 (31.0)	
Not applicable	1 (0.8)	0 (0)	1 (1.0)		0 (0)	1 (1.3)		0 (0)	1 (1.4)	
Respiratory comorbidities										
Yes	53 (43.8)	7 (35.0)	46 (45.5)	0.385	22 (47.8)	31 (41.3)	0.485	20 (40.0)	33 (46.5)	0.479
No	68 (56.2)	13 (65.0)	55 (54.5)		24 (52.2)	44 (58.7)		30 (60.0)	38 (53.5)	
Cardiovascular comorbidities										
Yes	24 (19.8)	5 (25.0)	18 (17.9)	0.455	10 (21.7)	13 (17.3)	0.549	7 (14.0)	16 (22.5)	0.239
No	97 (80.2)	15 (75.0)	83 (82.2)		36 (78.3)	62 (82.7)		43 (86.0)	55 (77.5)	
Surgical procedure										
Lobectomy	72 (59.5)	14 (70.0)	58 (57.4)	0.300	30 (65.2)	42 (56.0)	0.585	31 (62.0)	41 (57.7)	0.421
Segmentectomy	8 (6.6)	0 (0)	8 (7.92)		4 (8.7)	4 (5.3)		4 (8.0)	4 (5.6)	
Wedge resection	35 (28.9)	4 (20.0)	31 (30.7)		10 (21.7)	25 (33.3)		11 (22.0)	24 (33.8)	
Bilobectomy	5 (4.1)	2 (10.0)	3 (3.8)		2 (4.3)	3 (4.0)		3 (6.0)	2 (2.8)	
Pneumonectomy	1 (0.8)	0 (0)	1 (1.0)		0 (0)	1 (1.3)		1 (2.0)	0 (0)	
Lymph node dissection										
ND2	63 (52.1)	14 (70.0)	49 (48.0)	0.206	29 (63.0)	34 (45.3)	0.165	29 (59.0)	34 (47.9)	0.362
ND1	23 (19.0)	2 (10.0)	21 (21.2)		7 (15.2)	16 (21.3)		10 (20.0)	13 (18.3)	
ND0	35 (28.9)	4 (20.0)	31 (30.7)		10 (21.7)	25 (33.3)		11 (22.0)	24 (33.8)	
Curability										
R0	101 (83.5)	18 (90.0)	83 (82.2)	0.686	40 (87.0)	61 (81.3)	0.694	42 (84.0)	59 (83.1)	0.438
R1	11 (9.1)	1 (5.0)	10 (9.9)		3 (6.5)	8 (10.7)		3 (6.0)	8 (11.3)	
R2	9 (7.4)	1 (5.0)	8 (7.92)		3 (6.5)	6 (8.0)		5 (10.0)	4 (5.6)	
pT factor										
T1a	5 (4.1)	0 (0)	5 (5.0)	0.452	3 (6.5)	2 (2.7)	0.857	5 (10.0)	0 (0)	0.182
T1b	31 (25.6)	4 (20.0)	27 (26.7)		12 (26.1)	19 (25.3)		11 (22.0)	20 (28.2)	
T1c	24 (19.8)	2 (10.0)	22 (21.8)		8 (17.4)	16 (21.3)		10 (20.0)	14 (19.7)	
T2a	44 (36.4)	10 (50.0)	34 (33.7)		16 (34.8)	28 (37.3)		17 (34.0)	27 (38.0)	
T2b	5 (4.1)	2 (10.0)	3 (3.8)		3 (6.5)	2 (2.7)		3 (6.0)	2 (2.8)	
T3	7 (5.8)	1 (5.0)	6 (5.9)		2 (4.3)	5 (6.7)		2 (4.0)	5 (7.0)	
T4	5 (4.1)	1 (5.0)	4 (4.0)		2 (4.3)	3 (4.0)		2 (4.0)	3 (4.2)	
pN factor										
N0	61 (50.4)	13 (65.0)	48 (47.5)	0.487	29 (63.0)	32 (42.7)	0.244	31 (62.0)	30 (42.3)	0.196
N1	15 (12.4)	1 (5.0)	14 (13.9)		4 (8.7)	11 (14.7)		5 (10.0)	10 (14.1)	
N2	14 (11.6)	3 (15.0)	11 (10.9)		5 (10.9)	9 (12.0)		6 (12.0)	8 (11.3)	
N3	1 (0.8)	0 (0)	1 (1.0)		0 (0)	1 (1.3)		0 (0)	1 (1.4)	
NX	30 (24.8)	3 (15.0)	27 (26.7)		8 (17.4)	22 (29.3)		8 (16.0)	22 (31.0)	
Pathological stage										
I	75 (62.0)	12 (60.0)	63 (62.4)	0.696	29 (63.0)	46 (61.3)	0.868	33 (66.0)	42 (59.2)	0.651
II	23 (19.0)	4 (20.0)	19 (18.8)		9 (19.6)	11 (14.7)		8 (16.0)	15 (21.1)	
III	18 (14.9)	4 (20.0)	14 (13.9)		7 (15.2)	11 (14.7)		8 (16.0)	10 (14.1)	
IV	5 (4.1)	0 (0)	5 (5.0)		1 (2.2)	4 (5.3)		1 (2.0)	4 (5.6)	
Histology										
SCLC	50 (41.3)	8 (40.0)	42 (41.6)	0.984	15 (32.6)	35 (46.7)	0.022	18 (36.0)	32 (45.1)	0.064
LCNEC	36 (29.7)	6 (30.0)	30 (29.7)		20 (43.5)	16 (21.3)		21 (42.0)	15 (21.1)	
Combined SCLC	14 (11.6)	2 (10.0)	12 (11.9)		2 (4.3)	12 (16.0)		3 (6.0)	11 (15.5)	
Combined LCNEC	21 (17.4)	4 (20.0)	17 (16.8)		9 (19.6)	12 (16.0)		8 (16.0)	13 (18.3)	
Lymphatic invasion										
Present	80 (66.1)	13 (65.0)	67 (66.3)	0.276	29 (63.0)	51 (68.0)	0.502	31 (62.0)	49 (69.0)	0.206
Absent	35 (28.9)	3 (15.0)	32 (31.7)		15 (32.6)	20 (26.6)		17 (34.0)	18 (25.4)	
Not applicable	6 (5.0)	1 (5.0)	5 (5.0)		2 (4.3)	4 (5.3)		1 (2.0)	5 (7.0)	
Vascular invasion										
Present	81 (70.0)	16 (80.0)	64 (63.7)	0.041	33 (71.7)	48 (64.0)	0.343	33 (66.0)	48 (67.6)	0.619
Absent	35 (28.9)	3 (15.0)	32 (31.7)		11 (23.9)	24 (32.0)		16 (32.0)	19 (26.8)	
Not applicable	5 (4.1)	1 (5.0)	5 (5.0)		2 (4.3)	3 (4.0)		1 (2.0)	4 (5.6)	
Pleural invasion										
Present	52 (43.0)	10 (50.0)	42 (41.6)	0.533	21 (45.7)	31 (41.3)	0.733	21 (42.0)	31 (43.7)	0.751
Absent	67 (55.4)	10 (50.0)	57 (56.4)		25 (54.3)	42 (56.0)		29 (58.0)	38 (53.5)	
Not applicable	2 (1.7)	0 (0)	2 (2.0)		0 (0)	2 (2.7)		0 (0)	2 (2.8)	
Adjuvant chemotherapy										
Yes	66 (54.5)	10 (50.0)	56 (55.4)	0.590	24 (52.2)	42 (56.0)	0.567	29 (58.0)	37 (52.1)	0.494
No	53 (43.8)	10 (50.0)	43 (42.6)		22 (47.8)	31 (41.3)		20 (40.0)	33 (46.5)	
Not applicable	2 (1.7)	0 (0)	2 (2.0)		0 (0)	2 (2.7)		1 (2.0)	1 (1.4)	

Abbreviations: CAF, cancer‐associated fibroblast; LCNEC, large‐cell neuroendocrine carcinoma; PD‐L1, programmed death‐ligand 1; PDPN, podoplanin; SCLC, small‐cell lung carcinoma.

### Immunohistochemistry

**FIGURE 2:**
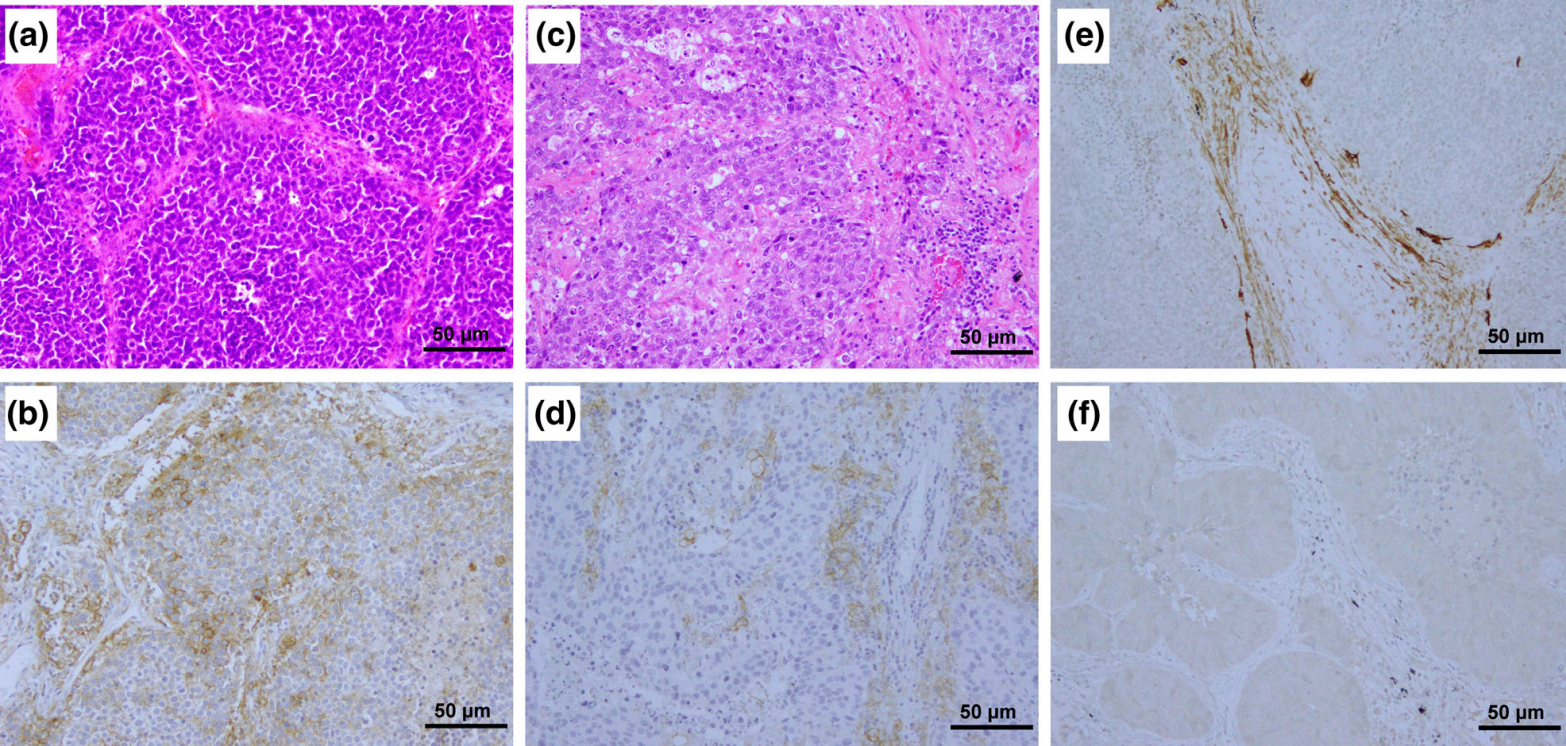
Representative images of tumoral programmed death‐ligand 1 (PD‐L1), stromal PD‐L1, and podoplanin (PDPN) expression in cancer‐associated fibroblasts (CAFs), (CAF‐PDPN) detected by immunohistochemistry (IHC) in high‐grade neuroendocrine carcinoma. (a) Hematoxylin and eosin staining (H&E) of small‐cell lung carcinoma (SCLC). (b) Tumoral PD‐L1 IHC of SCLC. The membranes of carcinoma cells stained positive for PD‐L1. (c) H&E of large‐cell neuroendocrine carcinoma (LCNEC). (d) Stromal PD‐L1 IHC of LCNEC. The tumor‐infiltrating immune cells in the stroma stained positive for stromal PD‐L1. (e) IHC of CAF‐PDPN‐positive case. The cell membranes of CAF stained positive for PDPN. (f) IHC of CAF‐PDPN‐negative case. The cell membranes of CAF stained negative for PDPN. Original magnification, ×200 for H&E and IHC.

Representative immunohistochemical staining of tumoral PD‐L1, stromal PD‐L1, and CAF‐PDPN are shown in Figure 2. Among 121 cases, the expression of tumoral PD‐L1, stromal PD‐L1, and CAF‐PDPN was detected in 20 (16.5%), 46 (38.0%), and 50 (41.3%) specimens, respectively.

### Correlations between CAF‐PDPN, tumoral PD‐L1, and stromal PD‐L1 expression levels

**FIGURE 3:**
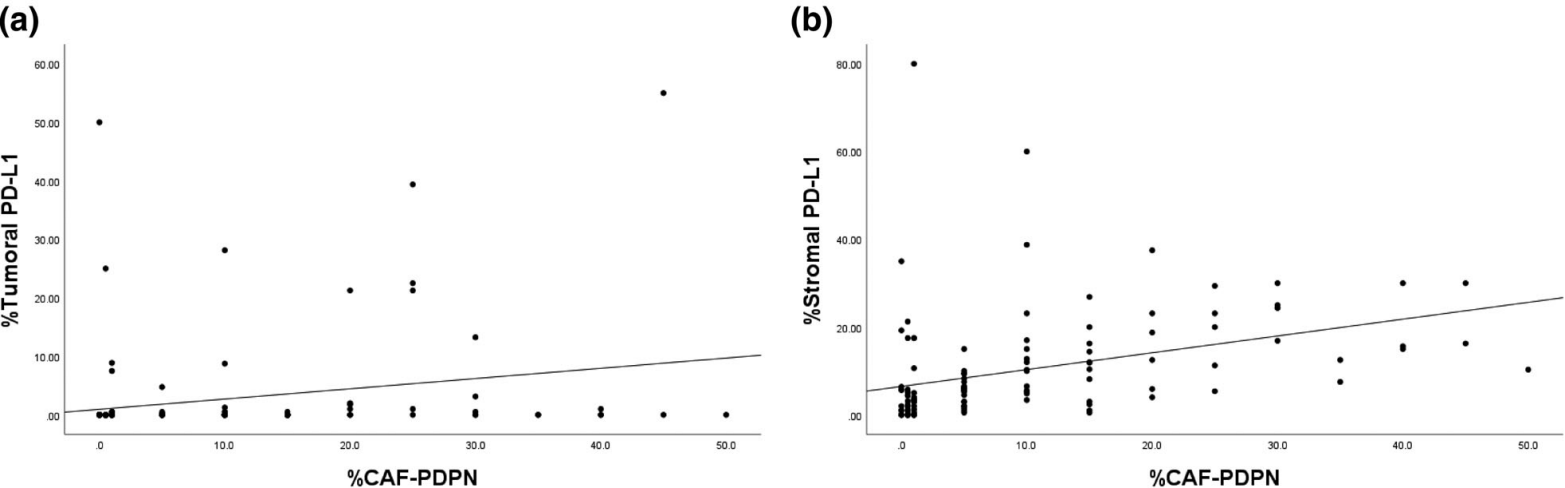
Scatter plots showing the correlation between (a) podoplanin (PDPN) in cancer‐associated fibroblasts (CAFs) (CAF‐PDPN) and tumoral programmed death‐ligand 1 (PD‐L1) expression and (b) between CAF‐PDPN and stromal PD‐L1 expression.

We tested for a correlation between CAF‐PDPN and tumoral PD‐L1 expression and between CAF‐PDPN and stromal PD‐L1 expression. CAF‐PDPN expression was weakly correlated with tumoral PD‐L1 expression (*ρ* = 0.299, *p* < 0.001) and moderately correlated with stromal PD‐L1 expression (*ρ* = 0.567, *p* < 0.001) (Figure 3).

### Survival analysis

**FIGURE 4:**
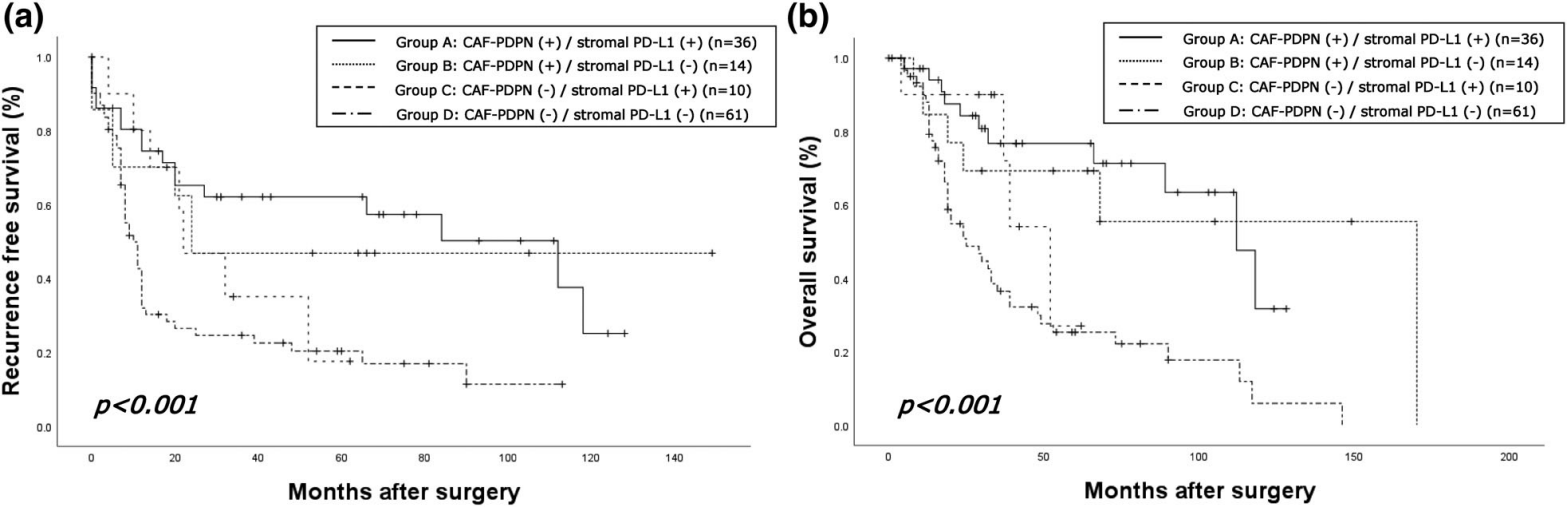
Kaplan–Meier curves for (a) recurrence‐free survival and (b) overall survival of high‐grade neuroendocrine carcinoma based on the combination of podoplanin (PDPN) in cancer‐associated fibroblasts (CAFs) (CAF‐PDPN) and stromal programmed death‐ligand 1 (PD‐L1) expression status. Group A: Positive expression of both CAF‐PDPN or stromal PD‐L1, CAF‐PDPN (+)/stromalPD‐L1 (+); Group B: Positive expression of CAF‐PDPN and negative expression of stromal PD‐L1, CAF‐PDPN (+)/stromal PD‐L1 (−); Group C: Negative expression of CAF‐PDPN and positive expression of stromal PD‐L1, CAF‐PDPN (−)/stromal PD‐L1 (+); Group D: Negative expression of both CAF‐PDPN and stromal PD‐L1, CAF‐PDPN (−)/stromal PD‐L1 (−). Statistical significance was determined by the log‐rank test.

**FIGURE 5:**
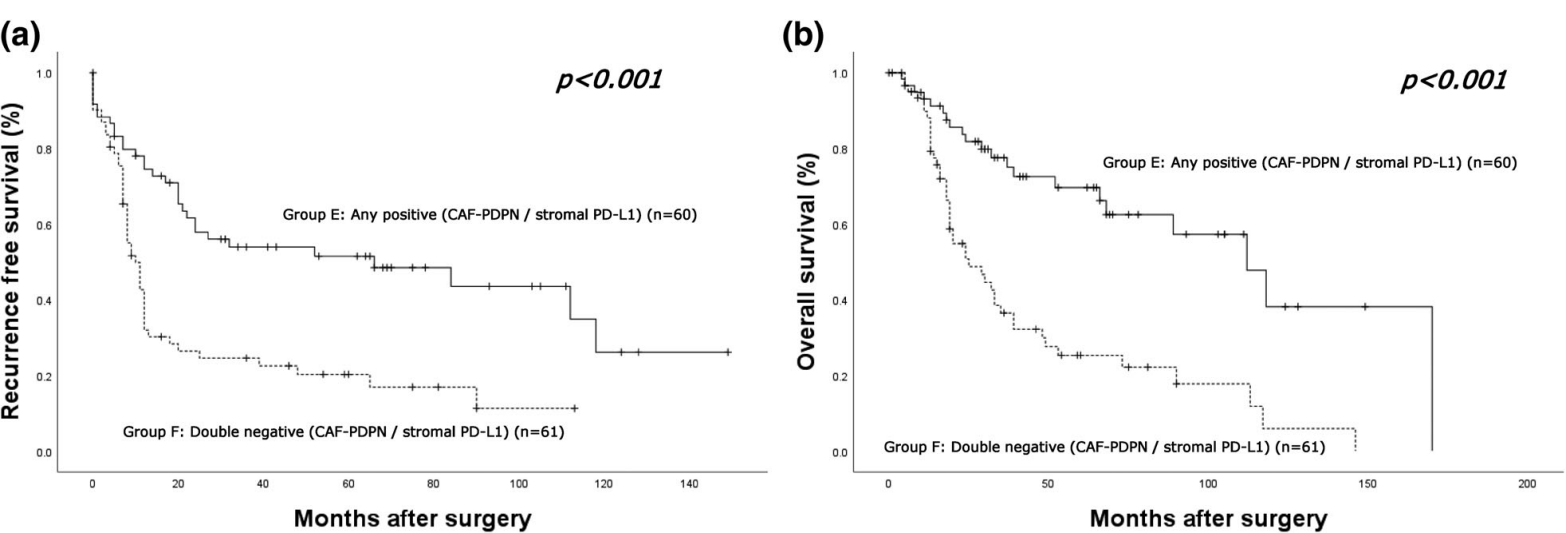
Kaplan–Meier curves for (a) recurrence‐free survival and (b) overall survival of high‐grade neuroendocrine carcinoma based on the combination of podoplanin (PDPN) in cancer‐associated fibroblasts (CAFs) (CAF‐PDPN) and stromal programmed death‐ligand 1 (PD‐L1) expression status. Group E: Positive expression of either CAF‐PDPN or stromal PD‐L1, any positive (CAF‐PDPN/stromal PD‐L1); Group F: Negative expression of both CAF‐PDPN and stromal PD‐L1, double negative (CAF‐PDPN/stromal PD‐L1). Statistical significance was determined by the log‐rank test.

Finally, we analyzed the outcomes of patients according to the immunohistochemical status of tumoral PD‐L1, stromal PD‐L1, and CAF‐PDPN. The median follow‐up time was 30.0 months (range, 0–170 months; mean, 42.0 months). There were 69 (57.0%) recurrence cases, consisting of 34 distant, 11 locoregional and distant, and 24 locoregional recurrences. Fifty patients died of lung cancer progression, and 15 died of other causes. Survival analyses were performed in groups combining the CAF‐PDPN and stromal PD‐L1 status and the CAF‐PDPN and tumoral PD‐L1 status. First, the patients were divided into four groups: Group A: CAF‐PDPN (+)/stromal PD‐L1 (+); Group B: CAF‐PDPN (+)/stromal PD‐L1 (−); Group C: CAF‐PDPN (−)/stromal PD‐L1 (+); Group D: CAF‐PDPN (−)/stromal PD‐L1 (−). The survival curves for the groups are shown in Figure 4. The 5‐year RFS rates of Group A, Group B, Group C, and Group D were 62.0%, 46.8%, 17.5%, and 20.2%, respectively, and the corresponding 5‐year OS rates were 76.6%, 69.2%, 27.0%, and 25.3%, respectively. The log‐rank test showed statistically significant differences among the groups in RFS (*p* < 0.001) and OS (*p* < 0.001). Next, all patients were divided into two groups (Group E: any positive CAF‐PDPN/stromal PD‐L1; Group F: double negative CAF‐PDPN/stromal PD‐L1). The survival curves for the groups are shown in Figure 5. The 5‐year RFS rates of Group E and Group F were 51.4% and 20.2%, respectively, and the corresponding 5‐year OS rates were 69.6% and 25.3%, respectively. The log‐rank test showed that Group E had more prolonged RFS (*p* < 0.001) and OS (*p* < 0.001) than Group F. The survival curves for all patients grouped by the presence or absence of CAF‐PDPN expression and stromal PD‐L1 expression, respectively, are shown in Figure S1. The 5‐year RFS rates of the CAF‐PDPN‐positive and ‐negative groups were 57.8% and 20.3%, respectively, and the corresponding 5‐year OS rates were 74.8% and 27.1%, respectively. The log‐rank test showed that the CAF‐PDPN‐positive group had longer RFS (*p* < 0.001) and OS (*p* < 0.001) compared with the CAF‐PDPN‐negative group (Figure S1A,B). The 5‐year RFS rates of the stromal PD‐L1‐positive and ‐negative groups were 52.5% and 25.2%, respectively, and the corresponding 5‐year OS rates were 68.7% and 34.1%, respectively. The log‐rank test showed that the stromal PD‐L1‐positive group had longer RFS (*p* = 0.002) and OS (*p* = 0.001) compared with the stromal PD‐L1‐negative group (Figure S1C,D).

### Univariate and multivariate analyses

Univariate analysis showed a statistically significant correlation between shorter RFS and negative status for both CAF‐PDPN and stromal PD‐L1 (hazard ratio [HR], 2.442; 95% confidence interval [CI], 1.533–3.890; *p* < 0.001), sublobar resection (HR, 1.950; 95% CI, 1.239–3.069; *p* = 0.003), ≥pN1 (HR, 2.487; 95% CI, 1.545–4.002; *p* < 0.001), pStage ≥ II (HR, 1.747; 95% CI, 1.120–2.725; *p* = 0.013), and the presence of lymphatic invasion (HR, 1.889; 95% CI, 1.108–3.221; *p* = 0.018). Multivariate analysis showed that negative status for both CAF‐PDPN and stromal PD‐L1 (HR, 2.343; 95% CI, 1.421–3.865; *p* < 0.001), sublobar resection (HR, 2.879; 95% CI, 1.681–4.932; *p* < 0.001), and ≥pN1 (HR, 3.008, 95% CI, 1.317–6.868; *p* = 0.009) were independent prognostic factors for RFS. Univariate analysis showed a statistically significant correlation between shorter OS and negative status for both CAF‐PDPN and stromal PD‐L1 (HR, 3.242; 95% CI, 1.899–5.534; *p* < 0.001), age ≥70 (HR, 1.846; 95% CI, 1.093–3.118; *p* = 0.020), ≥pN1 (HR, 2.005; 95% CI, 1.189–3.381; *p* = 0.008), and the presence of lymphatic invasion (HR, 2.189, 95% CI, 1.181–4.060; *p* = 0.011). Multivariate analysis showed that negative status for both CAF‐PDPN and stromal PD‐L1 (HR, 3.202; 95% CI, 1.817–5.644; *p* < 0.001), age ≥70 (HR, 2.012; 95% CI, 1.142–3.545; *p* = 0.016), and ≥pN1 (HR, 2.341; 95% CI, 1.291–4.245; *p* = 0.005) were independent prognostic factors for OS (Table 2).

**TABLE 2:** Univariate and multivariate analyses for recurrence‐free survival and overall survival.

Variables	Univariate analysis			Multivariate analysis		
	Hazard ratio	95% confidence interval	*p*‐value	Hazard ratio	95% confidence interval	*p*‐value
Univariate and multivariate analysis for RFS						
Age (year)						
≥70 versus <70	1.194	0.755–1.889	0.447			
Gender						
Female versus male	1.186	0.591–2.380	0.632			
Respiratory comorbidities						
Yes versus no	1.488	0.954–2.320	0.078			
Cardiovascular comorbidities						
Yes versus no	0.840	0.484–1.458	0.535			
Surgical procedure						
Sublobar versus lobectomy or more	1.950	1.239–3.069	0.003	2.879	1.681–4.932	<0.001
Histology						
SCLC or Combined SCLC versus others	0.858	0.549–1.341	0.502			
pT factor						
≥T2 versus T1	1.246	0.800–1.939	0.330			
pN factor						
≥N1 versus N0	2.487	1.545–4.002	<0.001	3.008	1.317–6.868	0.009
Pathological stage						
≥II versus I	1.747	1.120–2.725	0.013	1.251	0.571–2.743	0.575
Lymphatic invasion						
Present versus absent	1.889	1.108–3.221	0.018	1.601	0.901–2.846	0.109
Vascular invasion						
Present versus absent	1.264	0.761–2.098	0.365			
Pleural invasion						
Present versus absent	1.224	0.781–1.917	0.378			
Adjuvant chemotherapy						
No versus yes	1.115	0.708–1.756	0.638			
CAF‐PDPN and stromal PD‐L1 expression						
Double negative versus any positive	2.442	1.533–3.890	<0.001	2.343	1.421–3.865	<0.001
Univariate and multivariate analysis for OS						
Age (year)						
≥70 versus <70	1.846	1.093–3.118	0.020	2.012	1.142–3.545	0.016
Gender						
Female versus male	1.487	0.705–3.140	0.295			
Respiratory comorbidities						
Yes versus no	1.148	0.697–1.890	0.588			
Cardiovascular comorbidities						
Yes versus no	1.091	0.601–1.982	0.775			
Surgical procedure						
Sublobar versus Lobectomy or more	1.462	0.876–2.440	0.144			
Histology						
SCLC or Combined SCLC versus Others	1.024	0.624–1.681	0.926			
pT factor						
≥T2 versus T1	1.436	0.873–2.361	0.152			
pN factor						
≥N1 versus N0	2.005	1.189–3.381	0.008	2.341	1.291–4.245	0.005
Pathological stage						
≥II versus I	1.598	0.976–2.615	0.060			
Lymphatic invasion						
Present versus absent	2.189	1.181–4.060	0.011	1.653	0.859–3.180	0.132
Vascular invasion						
Present versus absent	1.324	0.760–2.304	0.320			
Pleural invasion						
Present versus absent	1.359	0.824–2.242	0.228			
Adjuvant chemotherapy						
No versus yes	1.323	0.800–2.187	0.274			
CAF‐PDPN and stromal PD‐L1 expression						
Double negative versus any positive	3.242	1.899–5.534	<0.001	3.202	1.817–5.644	<0.001

Abbreviations: CAF, cancer‐associated fibroblast; PDPN, podoplanin; PD‐L1, programmed death‐ligand 1; SCLC, small‐cell lung carcinoma.

## DISCUSSION

This study evaluated the presence, correlation, and prognostic value of CAF‐PDPN, tumoral PD‐L1, and stromal PD‐L1 expression in a multicenter cohort of patients with surgically resected HGNEC. Our findings show a positive correlation between CAF‐PDPN expression and tumoral/stromal PD‐L1 expression in resected HGNEC, and that the combination of these expression statuses can more accurately predict prognosis. In particular, the positive status of either CAF‐PDPN expression or stromal PD‐L1 expression was correlated with a favorable prognosis, indicating that they are independent prognostic biomarkers.

PDPN is a cell surface mucin‐like glycoprotein that is involved in the normal development of the lungs, kidneys, and lymphatic system but is overexpressed in several tumors and may also be important in the development and progression of malignant tumors.^12^ PDPN contributes to malignant tumor progression by regulating signaling that controls cell proliferation, differentiation, migration, invasion, epithelial–mesenchymal transition, and stemness.^12^ Overexpression of PDPN is observed not only in tumor cells but also in tumor stroma, including CAFs and immune cells. Several studies have demonstrated the role of PDPN‐positive CAF in the TME. Neri et al. showed that PDPN‐positive CAF activates the Rho‐ROCK pathway via its cytoplasmic domain, thereby facilitating cancer cell invasion.^17^ Suzuki et al. showed that PDPN‐positive CAF has higher expression of TGFB1, which is a known cytokine involved in M2 macrophage polarization and immunosuppression.^18^ Hu et al. conducted a meta‐analysis and found that PDPN‐positive CAFs were associated with poor prognosis in cholangiocarcinoma, breast cancer, NSCLC, and pancreatic cancer.^19^ These findings suggest that PDPN‐positive CAFs are associated with an immunosuppressive tumor microenvironment and tumor malignancy. However, the prognostic implications of the PDPN‐positive CAF in HGNEC were not fully understood. One report has focused on the prognostic implications of PDPN‐positive CAF in HGNEC. Takahashi et al. investigated the correlation between PDPN expression in CAF and the survival status of 115 patients with surgically resected HGNEC.^13^ In that study, patients with PDPN‐positive CAF had a more favorable prognosis than those with PDPN‐negative CAF. Because the results were contrary to their expectations, they are conducting additional experiments to test whether CAF‐expressing PDPN affects SCLC cell proliferation.^20^ In that study, the number of SCLC cells detected in co‐cultures with CAF overexpressing PDPN was decreased relative to the control. Conversely, suppressing PDPN expression via shRNA treatment in CAF increased the number of SCLC cells. In surgically resected human SCLC specimens, the frequency of geminin‐positive cancer cells was higher in the cases with PDPN‐positive CAF than in those with PDPN‐negative CAF. Therefore, CAF‐expressing PDPN inhibited the proliferation of SCLC cells, indicating that CAF‐expressing PDPN are a tumor‐suppressive stromal cell component in SCLC. In our study, PDPN‐positive CAFs were an independent favorable prognostic factor for OS and RFS according to the multivariate analyses, consistent with their reports.^13^ PDPN‐positive CAF may have tumor‐suppressive effects in HGNEC, but the exact molecular mechanism remains to be elucidated. In recent years, several studies have suggested the existence of subtypes of CAF that act as tumor suppressors.^21^, ^22^, ^23^, ^24^ CAF‐derived soluble factors, such as slit2 and asporin, and microRNAs, such as miR‐148b and miR‐139, have been proven to inhibit tumor progression.^25^, ^26^, ^27^, ^28^ We propose that such tumor suppressors and signaling pathways are involved in PDPN‐positive CAF in HGNEC. Further studies are needed to elucidate the molecular mechanisms of PDPN‐positive CAF function in HGNEC.

Although the relationship between CAF and PD‐L1 is not yet clear, it has been reported that CAFs induce PD‐L1 expression in various carcinomas. Inoue et al. showed that CAF increased PD‐L1 expression in lung adenocarcinoma cells via the secretion of soluble factors such as CXCL2.^29^ Sun et al. and Lou et al. showed that CAF‐derived IL‐8 upregulates PD‐L1 expression in gastric cancer cells through the STAT3 and NF‐κB pathways.^30^, ^31^ There are reports that CAF promoted PD‐L1 expression in tumor cells by activating AKT signaling.^32^, ^33^ Our study found a positive correlation between CAF‐PDPN expression and tumor/stromal PD‐L1 expression and a stronger positive correlation between CAF‐PDPN expression and stromal PD‐L1 expression in resected HGNEC. The results suggest that PDPN‐positive CAF in the TME may indirectly affect tumor immunity via increased PD‐L1 expression in the stromal immune cells. To our knowledge, this is the first report investigating the correlation between CAF‐PDPN and tumoral/stromal PD‐L1 expression in HGNEC. In addition, dividing patients into four groups based on the combination of CAF‐PDPN and stromal PD‐L1 expression resulted in a differential prognosis. Based on the results, the predictive prognostic value was different between two groups of patients: one positive for expression of either CAF‐PDPN or stromal PD‐L1, and the other negative for expression of both CAF‐PDPN and stromal PD‐L1. This is considered to have clinical significance.

There are some limitations to this study. First, it is a retrospective, nonrandomized study and limited to surgical resection cases, which may have selection bias. The limited sample size due to the rarity of the disease has not allowed for comparisons in validation cohorts or control groups. It may have been biased because administrative decisions regarding surgical procedures, postoperative adjuvant therapy, and follow‐up intervals were left to each participating center. Second, the data collection period was from 2005 to 2022, during which lung cancer treatment changed in terms of diagnosis, staging, surgery, and radiation therapy. Third, no standardized protocols for evaluating CAF‐PDPN and tumoral/stromal PD‐L1 exist. Different results may be obtained depending on the antibodies, staining protocols, cutoff values, and scoring methods. Takada et al. showed that there was no difference in expression between SP142, which is designed as an antibody to evaluate PD‐L1 expression in immune cells, and E1L3N, which was not so designed.^34^ The same tendency was observed in this study, but a proper evaluation could not be performed. Fourth, the pStage of pNX cases was determined by presuming N0, which may not have resulted in correct staging.

In conclusion, this study shows that CAF‐PDPN expression correlates with tumoral/stromal PD‐L1 expression and has positive prognostic implications in patients with surgically resected HGNEC. CAF‐PDPN and stromal PD‐L1 expression may be prognostic factors for HGNEC and may provide important information for developing new therapeutic strategies.

## AUTHOR CONTRIBUTIONS

All authors have made significant contributions to this paper. *Conceptualization*: Tomohiro Haruki, Tatsuya Miyamoto, Shinji Matsui, Yuki Oshima, and Yoshihisa Umekita. *Data curation*: Tatsuya Miyamoto, Karen Makishima, Shinji Matsui, Yuki Oshima, and Tomohiro Haruki. *Formal analysis*: Tatsuya Miyamoto and Karen Makishima. *Supervision*: Hiroshige Nakamura and Yoshihisa Umekita. *Writing—original draft preparation*: Tatsuya Miyamoto; *Writing—review and editing*: Tomohiro Haruki, and Yoshihisa Umekita.

## FUNDING INFORMATION

This work was supported by the Japan Society for the Promotion of Science (JSPS KAKENHI grant number JP 22K09002).

## CONFLICT OF INTEREST STATEMENT

The authors declare no conflicts of interest.

## Supporting information


**Figure S1.** Kaplan–Meier curves for (A, C) recurrence‐free survival and (B, D) overall survival of high‐grade neuroendocrine carcinoma based on the podoplanin (PDPN) in cancer‐associated fibroblasts (CAFs) (CAF‐PDPN) and stromal programmed death‐ligand 1 (PD‐L1) expression status. Statistical significance was determined by the log‐rank test.

## Data Availability

The authors confirm that the data supporting the findings of this study are available within the article and its Supporting Information.
