# Supplementary material for: The prognostic implications of podoplanin in cancer‐associated fibroblasts and PD‐L1 expression in high‐grade neuroendocrine carcinoma of the lung
Source: Thorac Cancer. 2024 Nov 2;15(36):2519–29. doi: 10.1111/1759-7714.15477 (PMC11669446; doi:10.1111/1759-7714.15477)
Supplement: Supplementary file 1 — Figure S1. Kaplan–Meier curves for (A, C) recurrence‐free survival and (B, D) overall survival of high‐grade neuroendocrine carcinoma based on the podoplanin (PDPN) in cancer‐associated fibroblasts (CAFs) (CAF‐PDPN) and stromal programmed death‐ligand 1 (PD‐L1) expression status. Statistical significance was determined by the log‐rank test. [file TCA-15-2519-s001.pptx]

## Slide 1
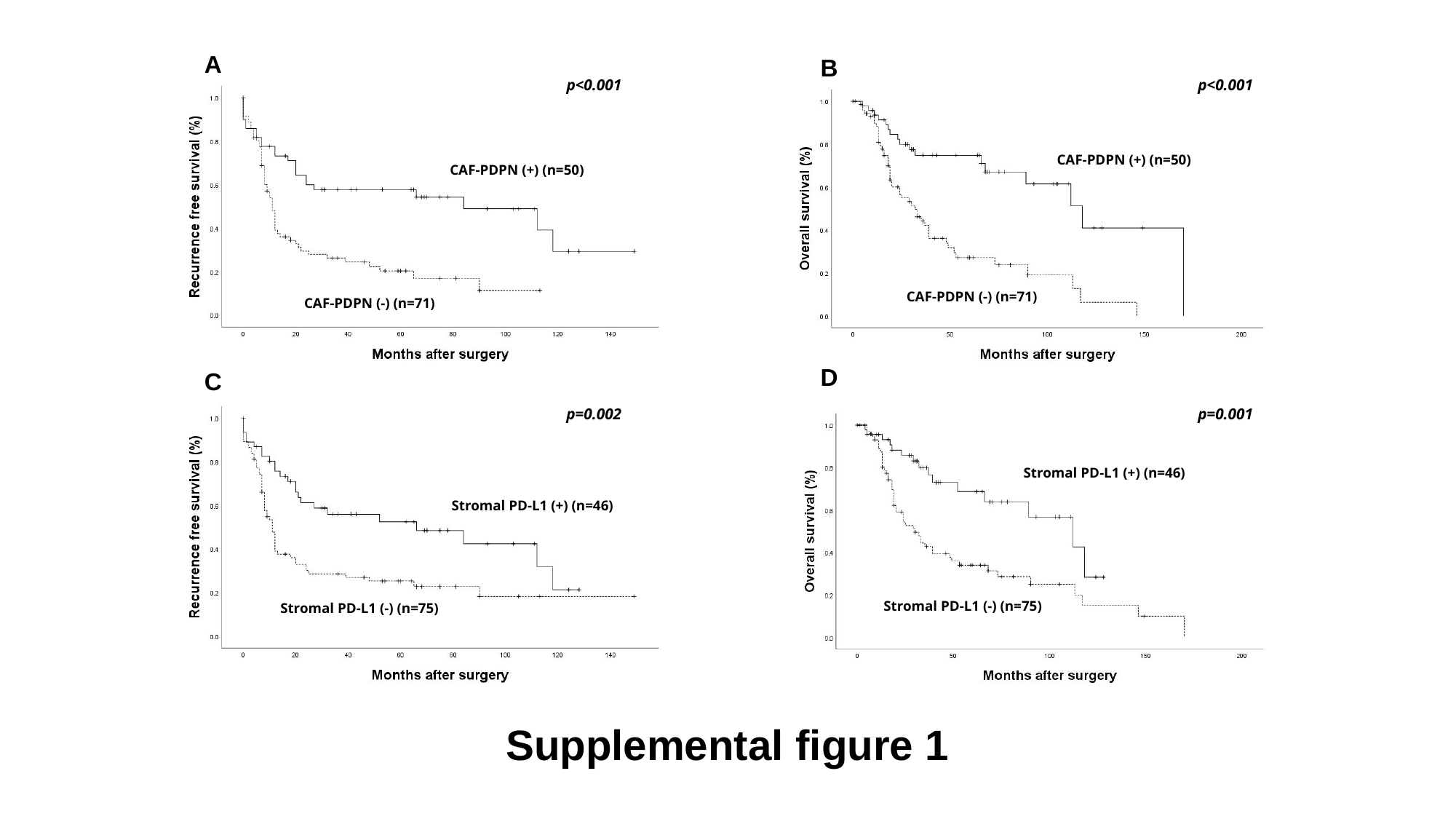

A
B
p<0.001
p<0.001
CAF-PDPN (+) (n=50)
CAF-PDPN (+) (n=50)
CAF-PDPN (-) (n=71)
CAF-PDPN (-) (n=71)
D
C
p=0.002
p=0.001
Stromal PD-L1 (+) (n=46)
Stromal PD-L1 (+) (n=46)
Stromal PD-L1 (-) (n=75)
Stromal PD-L1 (-) (n=75)
Supplemental figure 1
